# Mental health symptoms and sleep quality of asymptomatic/mild SARS‐CoV‐2 infected individuals during the Omicron wave of the COVID‐19 pandemic in Shanghai China

**DOI:** 10.1002/brb3.2803

**Published:** 2022-11-03

**Authors:** Zhenghua Hou, Yingzi Huang, Shaolei Ma, Hui Feng, Cuiping Fu, Han Li, Yuexing Yuan, Yonggui Yuan

**Affiliations:** ^1^ Department of Psychosomatics & Psychiatry, Affiliated Zhongda Hospital, School of Medicine Southeast University Nanjing China; ^2^ Department of Critical Care Medicine, Zhongda Hospital, School of Medicine Southeast University Nanjing China; ^3^ Department of Emergency and Critical Care Medicine, Zhongda Hospital, School of Medicine Southeast University Nanjing China; ^4^ Department of Traditional Chinese Medicine, Zhongda Hospital, School of Medicine Southeast University Nanjing China; ^5^ Department of Respiratory and Critical Care Medicine, Zhongda Hospital, School of Medicine Southeast University Nanjing China; ^6^ Department of Pediatrics, Zhongda Hospital, School of Medicine Southeast University Nanjing China; ^7^ Department of Endocrinology, Zhongda Hospital, Institute of Diabetes School of Medicine Southeast University Nanjing China

**Keywords:** COVID‐19, anxiety, depression, asymptomatic, mild infection, Omicron

## Abstract

**Objective:**

To investigate mental health symptoms (anxiety, depression, and sleep status) and their associated factors among people infected with the SARS‐CoV‐2 omicron variant during the quarantine period in Shanghai.

**Methods:**

To investigate the mental health symptoms among participants with SARS‐CoV‐2 omicron infection, an anonymous online survey questionnaire was used. The survey panel included the 9‐item Patient Health Questionnaire‐9 (PHQ‐9), 7‐item Generalized Anxiety Disorder Scale (GAD‐7), Pittsburgh Sleep Quality Index (PSQI), and 22‐item Ruminative Responses Scale (RRS). Group comparisons and correlation analyses were employed to explore the epidemiological characteristics of patients and factors related to depression and anxiety symptoms.

**Results:**

A total of 960 participants completed the survey. Of the total respondents, 583 participants (60.7%) were male, and the mean (SD) age was 34.33 (9.21) years (95% CI: 33.74–34.91). The prevalence of depressive and anxiety symptoms among the participants was 13.7% (*n* = 151, 95% CI: 11.6%–15.7%) and 8.6% (*n* = 90, 95% CI: 6.9%–10.3%), respectively. Age‐stratified analysis showed that the prevalence of anxiety among the 36‐ to 45‐year‐old group (12.9%; *n* = 35, 8.9%–16.9%) was significantly higher than that of the 18‐ to 15‐year‐old group (7.4%; *n* = 42, 5.3%–9.6%, *p* = .011). Spearman's correlation analyses showed that rumination (assessed by the RRS) was significantly and positively correlated with depression (rho = .706, *p* < .001) and anxiety symptoms (rho = .758, *p* < .001).

**Conclusion:**

The results suggest that female and middle‐aged populations manifest higher susceptibility to mental health distress during the current Omicron wave of the COVID‐19 pandemic. Population‐specific psychological crisis intervention is warranted to improve the quality of epidemic prevention methods and to promote the mental well‐being of the public.

## 1 INTRODUCTION

Before the emergence of coronavirus disease 2019 (COVID‐19), depressive and anxiety disorders were leading contributors to the global health‐related burden (GBD 2019 Mental Disorders Collaborators, 2022). The outbreak of the COVID‐19 pandemic has affected a large proportion of the world's population via social restrictions, lockdowns, office and school closures, and declining incomes, further exacerbating the prevalence of depression and anxiety symptoms (COVID‐19 Mental Disorders Collaborators, 2021). A study from COVID‐19 Mental Disorders Collaborators showed that the pandemic was associated with an increase in the global prevalence of major depressive disorder (increase of 27.6%) and anxiety disorders (increase of 25.6%) (COVID‐19 Mental Disorders Collaborators, 2021).

An international study (Wang et al., 2021) also suggested that the Poland and the Philippines were the two countries with the highest levels of anxiety, depression, and stress, whereas Vietnam had the lowest mean levels of anxiety, depression, and stress. Several studies from Asian regions (Japan, South Korea, and Singapore) also reported an increased prevalence of anxiety and depressive symptoms during the COVID‐19 pandemic (Park et al., 2021; Teo et al., 2021; Ueda et al., 2020). Additionally, researchers in Bangladesh have investigated the mental health status of the general population, medical and nursing population, college students, and adolescents during the epidemic, providing valuable information for further understanding the impact of the epidemic in Asia (Das et al., 2021; Islam et al., 2021a; Islam et al., 2021b; Repon et al., 2021).

In China, an online survey investigating the general population of 34 province‐level regions during the COVID‐19 outbreak suggested that 27.9% of respondents had depression symptoms of, 31.6% had anxiety symptoms, and 29.2% had symptoms of insomnia (Shi et al., 2020). Elevating the priority of mental health care interventions is imperative for promoting mental wellbeing and reducing the burden of disease.

In February, 2022, a wave of severe acute respiratory syndrome coronavirus 2 (SARS‐CoV‐2) infection spread rapidly in Shanghai, which was identified as a sublineage of the Omicron variant (BA2.2) of SARS‐CoV‐2 (Zhang et al., 2022). The Omicron variant strain is relatively less virulent but highly infectious and has a greater impact on the life and health of the unvaccinated elderly population (Guo et al., 2022; He et al., 2021). Shanghai has taken active and effective life‐saving efforts, including large‐scale nucleic acid screening, quarantine of infected cases in shelter hospitals, and lockdown of severely infected districts. Strict control strategies substantially reduce the number of infections and provide appropriate treatment for severe cases.

Additionally, local residents may experience inconveniences in daily life due to prevention and control measures, such as restricted outdoor recreation and reduced interpersonal communication (Kohrt, 2021; Steardo et al., 2020). These may act as detrimental factors of mental health, increasing susceptibility to depression, anxiety symptoms, and sleep disturbance (The Lancet, 2021). According to a previous study (COVID‐19 Mental Disorders Collaborators, 2021), prevention and control measures may significantly negatively affect the mental health in women and young people significantly. It is important to obtain the up‐to‐date mental health information of infected individuals so that the mental health system can adjust interventions in a timely manner.

## 2 METHODS

### 2.1 Study design

This cross‐sectional online survey with a nonprobability sampling design was conducted from April 5, 2022, to May 18, 2022. This study was conducted in accordance with the American Association for Public Opinion Research reporting guidelines and was approved by the ethics committee of the Affiliated Zhongda Hospital of Southeast University. Online written informed consent was obtained before participation. Before starting the questionnaire, subjects were screened to exclude people with past or current anxiety, depression, or sleep disorders, as well as those with a family history of mental disorders or alcohol and sleeping pill abuse.

We surveyed the people infected with the SARS‐CoV‐2 Omicron variant (frontline medical staff were excluded) during the quarantine period in Lingang Shelter hospital, Pudong Shanghai. The respondents anonymously completed the questionnaire via an online survey platform (wjx.cn) during the breaks, which also reduced unnecessary contact. The Quick Response code (QR code) linking the online questionnaire was presented to the individuals, and necessary explanations and guidance were provided (e.g., information about confidentiality) during the survey. PASS software was used to estimate the minimum sample size, the power (1‐β) was set as .80, and the type‐I error rate (α) was .05. Assuming a completion rate of approximately 80%, the final sample size needs to exceed 500 to obtain sufficient statistical power. Thirty‐six of the submitted questionnaires did not meet the quality requirements, so the effective completion rate was 96%.

### 2.2 Measurements

Participants completed a self‐designed online survey composed of five sections: demographic information (including gender, age, level of education, marital status), the Chinese version of the 9‐item Patient Health Questionnaire (PHQ‐9) (Spitzer et al., 1999), the 7‐item Generalized Anxiety Disorder Scale (GAD‐7) (Lowe et al., 2008), the Pittsburgh Sleep Quality Index (PSQI) (Tsai et al., 2005), and the 22‐item Ruminative Responses Scale (RRS) (Nolen‐Hoeksema et al., 2008). The survey took approximately 10–15 min to complete.

The PHQ‐9 and GAD‐7 offer clinicians self‐administered screening and diagnostic tools for mental health disorders, and these tools have been field‐tested in clinical settings (Spitzer et al., 1999; Spitzer et al., 2006; Zhang et al., 2013). The screening methods were concise and user‐friendly, improving the recognition rate of depression and anxiety and facilitating diagnosis and treatment (https://www.phqscreeners.com). Each item on these 2 measures asked about the respondent's experience in the past 2 weeks and was scored on a 4‐point scale (0 = not at all; 1 = some of the time; 2 = more than half the time; 3 = nearly every day). The total scores of the PHQ‐9 and GAD‐7 ranged from 0–27 to 0–21, respectively. According to previous studies (Kroenke, 2002; Liu et al., 2021; Lowe et al., 2008), a cutoff value of 10 points demonstrated good reliability and validity for indicating clinical depression or anxiety symptoms.

The PSQI is widely used to evaluate the sleep quality of subjects in the last month. This index includes 18 items across 7 components (sleep quality, sleep latency, sleep duration, sleep efficiency, sleep disturbance, use of sleeping medication, and daytime dysfunction). Each component was scored on a 0–3 scale, and the total PSQI score ranged from 0 to 21, with high scores indicating poor sleep quality.

The RRS was compiled by Pro. Nolen‐Hoeksema of Yale University (Nolen‐Hoeksema et al., 2008) and was translated and introduced by Han X and Yang H in 2009 (Han & Yang, 2009). The scale contains 22 items and is divided into three dimensions: symptom rumination, self‐blame brooding, and reflective pondering. Each item was scored on a 1–4 scale (1 = never; 2 = sometimes; 3 = often; 4 = always), with higher scores indicating more severe rumination tendency.

### 2.3 Statistical analyses

All of the statistical analyses were performed using SPSS statistical software (IBM Corp. Released 2012. IBM SPSS Statistics for Windows, Version 21.0. Armonk, NY: IBM Corp.). Descriptive statistics, including the mean, standard deviation (SD) and 95% confidence interval (95% CI), were calculated to represent demographic characteristics (age, sex, education level, and marital status). A chi‐square test was employed to compare the prevalence of self‐reported depression and anxiety symptoms in different subpopulations. The Mann‐Whitney *U* test or one‐way analysis of variance (ANOVA) was used to explore the group differences in PSQI/RRS scores, and post hoc tests were performed with Bonferroni correction. To reveal potential factors affecting anxiety, depressive symptoms and sleep quality, binary/multiple logistic regression analyses and Pearson's/Spearman's correlation analyses were performed. A two‐tailed *p* < .05 was considered to indicate statistical significance.

## 3 RESULTS

### 3.1 Demographic characteristics

Data from 960 participants were eligible for statistical analysis. Among all the respondents, 583 participants (60.7%) were male, and the mean (SD) age was 34.33 (9.21) years (95% CI: 33.74–34.91); 565 participants (58.9%) were aged 18 to 35 years; and 272 participants (28.3%) were aged 36 to 45 years. Of the total participants, 606 (63.1%) were married and 54 (5.6%) were divorced; 376 (39.2%) had a junior school education, 200 (20.8%) had a high school education, and 283 (29.5%) had a university degree or higher.

### 3.2 Prevalence of symptoms of depression and anxiety

**FIGURE 1:**
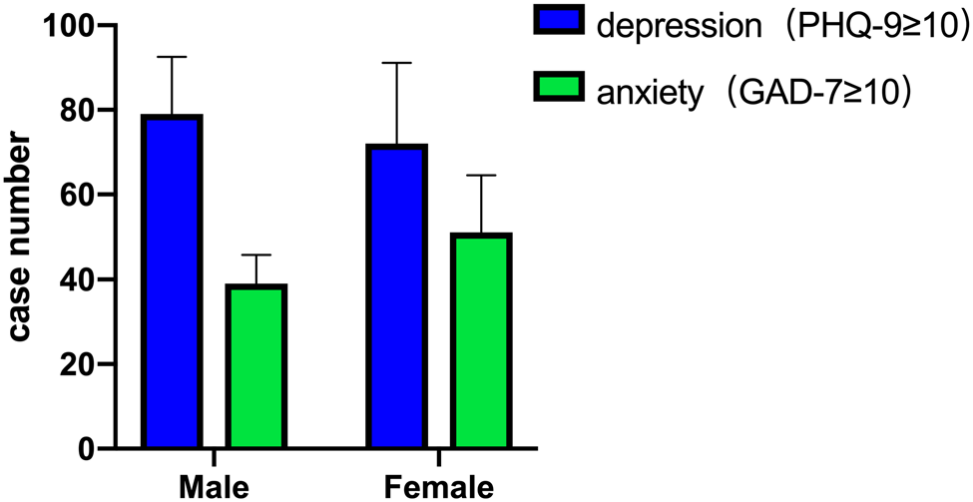
Comparison of cases with depression and anxiety symptom between male and female groups

The prevalence of depressive and anxiety symptoms among the total participants was 13.7% (*n* = 151, 95% CI: 11.6%−15.7%) and 8.6% (*n* = 90, 95% CI: 6.9%−10.3%), respectively. Group comparison showed that females had a higher prevalence of anxiety (13.53%, *n* = 51, 95% CI: 10.1%−17.0%), depression (19.10%, *n* = 72, 95% CI: 15.1–23.1), and rumination scores (33.75 ± 12.50) than males, while males had lower PSQI scores (sleep quality) than females (Table 1, Figure 1).

**TABLE 1:** Analysis of anxiety, depression, sleep quality, and rumination by gender

Group	Male (*n* = 583)	Female (*n* = 377)	χ^2^/*U*/*p*
Depression symptom No. (%, 95% CI)	79 (13.55, 10.8–16.3)	72 (19.10, 15.1–23.1)	χ^2^ = 5.316, *p* = .021
Anxiety symptom No. (%, 95% CI)	39 (6.69, 4.7–8.7)	51 (13.53, 10.1–17.0)	χ^2^ = 12.60, *p* < .001
RRS‐22	31.93 ± 11.06	33.75 ± 12.50	*U* = 100,836, *p* = .030
PSQI	5.303 ± 3.146	6.098 ± 3.507	*U* = 95,646, *p* < .001

Abbreviations: 95% CI, 95% confidence interval; PSQI, the Pittsburgh Sleep Quality Index; RRS‐22, the 22‐item Ruminative Responses Scale; U, Mann–Whitney *U* test; χ^2^, Pearson's chi‐square value.

Marital status was stratified into three groups: unmarried, married, and divorced. There was no significant difference in the prevalence of depression and anxiety among the three groups. Additionally, the PSQI scores of the unmarried group (5.064 ± 3.142) were significantly lower than those of the married (5.826 ± 3.343, Bonferroni *p =* .003) and divorced groups (6.315 ± 3.565, Bonferroni *p* = .031), and there was no significant difference in PSQI scores between the married and divorced groups (*p* > .05). Both the married (33.12 ± 11.85, *p =* .025) and divorced groups (34.91 ± 12.04, *p =* .035) exhibited higher RRS scores than the unmarried group (31.28 ± 11.12) (Table 2).

**TABLE 2:** Analysis of anxiety, depression, sleep quality, and rumination stratified by marital status

Group	Unmarried (*n* = 300)	Married (*n* = 606)	Divorced (*n* = 54)	χ^2^/*F*/*p*
Depression symptom No. (%, 95% CI)	45 (15, 10.9–19.1)	99 (16.3, 13.4–19.3)	7 (13.0, 3.7–22.2)	χ^2^ = 0.601, *p* = .741
Anxiety symptom No. (%, 95% CI)	19 (6.3, 3.6–9.1)	66 (10.9, 8.4–13.4)	5 (9.3, 1.3–17.2)	χ^2^ = 4.907, *p* = .086
RRS‐22	31.28 ± 11.12	33.12 ± 11.85	34.91 ± 12.04	*F* = 3.587, *p* = .028
PSQI	5.064 ± 3.142	5.826 ± 3.343	6.315 ± 3.565	*F* = 6.654, *p* = .001
PSQI Unmarried vs. Married	Bonferroni *p =* .003			
PSQI Unmarried vs. Divorced	Bonferroni *p* = .031			

Abbreviations: 95% CI, 95% confidence interval; PSQI, the Pittsburgh Sleep Quality Index; RRS‐22, the 22‐item Ruminative Responses Scale; χ^2^, Pearson's chi‐square value.

Age‐stratified analyses showed that the prevalence of anxiety in the 36‐ to 45‐year‐old group (12.9%, *n* = 35, 8.9%−16.9%) was significantly higher than that in the 18‐ to 35‐year‐old group (7.4%, *n* = 42, 5.3%−9.6%, *p* = .011), and the PSQI score in the **≥**46‐year‐old group (6.447 ± 3.694) was significantly higher than that in the 18‐ to 35‐year‐old group (5.368 ± 3.150, *p* = .003). There were no significant differences in depression, anxiety, RRS, or PSQI scores among the remaining subgroups (Table 3).

**TABLE 3:** Analysis of depression, anxiety, sleep quality, and rumination stratified by age

Group	18 ≤ Age ≥ 35 (*n* = 565)	36 ≤ Age ≥ 45 (*n* = 272)	Age ≥ 46 (*n* = 123)	χ^2^/*F*/*p*
Depression symptom No. (%, 95% CI)	86 (14.8, 11.9–17.7)	44 (16.2, 11.8–20.6)	21 (17.1, 10.3–23.8)	χ^2^ = 0.319, *p* = .853
Anxiety symptom No. (%, 95% CI)	42 (7.4, 5.3–9.6)	35 (12.9, 8.9–16.9)	13 (10.6, 5.1–16.1)	χ^2^ = 6.618, *p* = .037
RRS‐22	31.96 ± 10.97	33.26 ± 12.14	34.37 ± 13.45	*F* = 2.702/0.068
PSQI	5.368 ± 3.150	5.754 ± 3.406	6.447 ± 3.694	*F* = 5.741/0.003
PSQI group 1 vs. 3	Bonferroni *p* = .003			
GAD‐7 group 1 vs. 2	χ^2^ = 6.491, *p* = .011			

Abbreviations: 95% CI, 95% confidence interval; GAD‐7, the 7‐item Generalized Anxiety Disorder scale; PSQI, the Pittsburgh Sleep Quality Index; RRS‐22, the 22‐item Ruminative Responses Scale; χ^2^, Pearson's chi‐square value, group1: 18 ≤ Age ≥ 35, group 2: 36 ≤ Age ≥ 45, group 3:

Stratified analysis by education level revealed that there were no significant differences in the prevalence of depression and anxiety or RRS and PSQI scores among the groups (all *p* > .05). However, the prevalence of depression and anxiety in the lower educational level group (≤6 years) was higher than that in the other groups (education level: 9, 12, and ≥16 years) (Table 4).

**TABLE 4:** Analysis of anxiety, depression, sleep quality, and rumination by education level

Education	≤ 6 years (*n* = 62)	9 years (*n* = 376)	12 years (*n* = 239)	≥16 years (*n* = 283)	χ^2^/*F*/*p*
Depression symptom No. (%, 95% CI)	14 (22.6, 11.9–33.3)	62 (16.5, 12.7–20.3)	39 (16.3, 11.6–21.0)	36 (12.7, 8.8–16.6)	χ^2^ = 4.354, *p* = .226
Anxiety symptom No. (%, 95% CI)	10 (16.1, 6.7–25.5)	38 (10.1, 7.0–13.2)	20 (8.4, 4.8–11.9)	22 (7.8, 4.6–10.9)	χ^2^ = 4.705, *p* = .195
PSQI	6.065 ± 3.343	5.765 ± 3.178	5.483 ± 3.407	5.431 ± 3.402	*F* = 1.054, *p* = .368
RRS‐22	33.45 ± 15.34	33.21 ± 12.13	32.28 ± 11.23	32.01 ± 10.45	*F* = 0.745, *p* = .525

Abbreviations: 95% CI, 95% confidence interval; PSQI, the Pittsburgh Sleep Quality Index; RRS‐22, the 22‐item Ruminative Responses Scale; χ^2^, Pearson's chi‐square value.

### 3.3 Correlation and regression analyses

**FIGURE 2:**
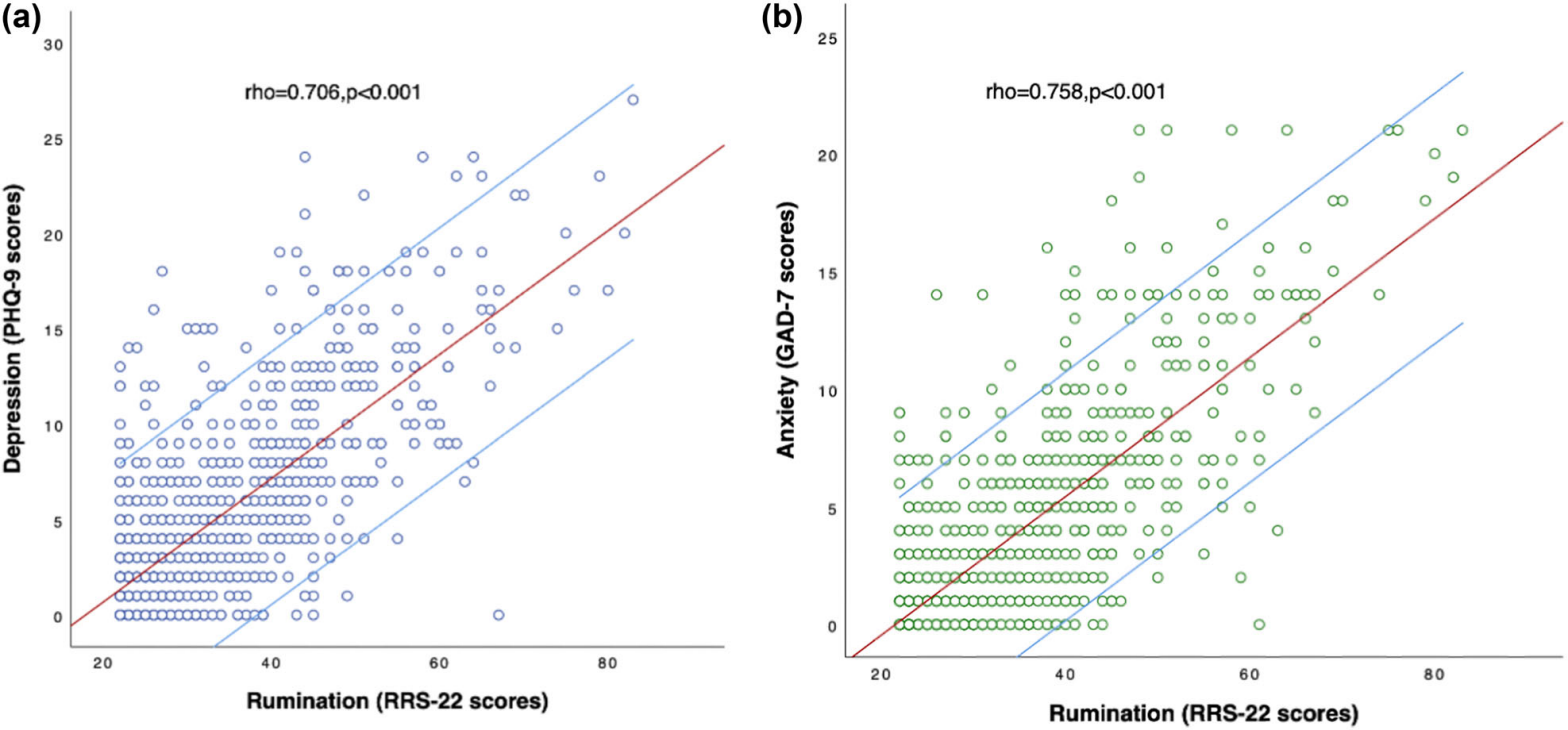
Spearman's correlation analyses between the rumination (RSS‐22 score) and depression /anxiety symptoms. *Note*: PHQ‐9, the 9‐item Patient Health Questionnaire; GAD‐7, the 7‐item Generalized Anxiety Disorder scale; RRS‐22, the 22‐item Ruminative Responses Scale; rho, Spearman's correlation coefficient. *p* Values were corrected with Bonferroni.

Spearman's correlation analyses showed that rumination was significantly positively correlated with depression (rho = .706, *p* < .001, Bonferroni corrected) and anxiety symptoms (rho = .758, *p* < .001, Bonferroni corrected) (Figure 2). With age, sex, level of education, and marital status as dependent variables and anxiety and depression symptoms as independent variables, no statistically significant symptom‐related risk factors were determined by binary logistic regression analysis.

## 4 DISCUSSION

Based on an anonymous online questionnaire survey, the present study preliminarily investigated the prevalence of mental health status (depression, anxiety, and sleep quality) and potential influencing factors among asymptomatic/mild infected individuals during the Omicron wave of the COVID‐19 pandemic in Shanghai, China. The online survey revealed that the rate of self‐reported mental symptoms among respondents with asymptomatic/mild infection was 15.7% for depressive symptoms and 9.4% for anxiety symptoms. The current study further estimated that the prevalence of depression and anxiety in women was significantly higher than that in men, which is consistent with previous studies (Baxter et al., 2016; Global Burden of Disease Study, 2015). Interestingly, the results also revealed that compared with men, women exhibited higher rumination scores and poorer sleep quality, which may also become susceptibility factors for mental health, making them more likely to develop depression and anxiety symptoms during the pandemic.

Before the outbreak of COVID‐19, the results from GBD 2019 (GBD 2019 Mental Disorders Collaborators, 2022) revealed that depression and anxiety mental disorders remained among the leading contributors to disease burden worldwide, with no significant evidence of a downward trend in the global burden since 1990. The COVID‐19 Mental Disorders Collaborators (COVID‐19 Mental Disorders Collaborators, 2021) estimated that the global prevalence of depression and anxiety increased by 27.6% (total prevalence: 3.2%) and 25.6% (total prevalence: 4.8%) in the general population due to the COVID‐19 pandemic, and the corresponding disability‐adjusted life‐years were also increased. The emergence of the COVID‐19 pandemic has brought adverse determinants of mental health worldwide, such as community lockdown, quarantine, close of work and study, and a decline in income (Gavin et al., 2021; Kohrt, 2021). These issues further exacerbate the mental health symptoms.

In China, the National Mental Health Survey (Huang et al., 2019) before the pandemic estimated that the 12‐month prevalence rates of depressive disorders and anxiety disorders in the general population were 3.6% and 5.0%, respectively. However, during the COVID‐19 pandemic, a recent survey (Shi et al., 2020) reported that the prevalence of mental health symptoms among the general population in China increased dramatically by 27.9% for depression, 31.6% for anxiety and 29.2% for insomnia. It is worth noting that in a previous survey of people with micron infection, the prevalence of depression and anxiety symptoms did not increase as much as in a previous study (Shi et al., 2020), but was slightly higher than results from other studies before the epidemic (approximate rate for depression: 6.0%; approximate rate for anxiety: 5.3%) (Spitzer et al., 2006; Yu et al., 2018; Zhou et al., 2014). The possible reasons are as follows. First, the investigation period of Shi et al. (2020) was from February to March 2020, when the epidemic had just broken out, and the infections were severe, which had a greater negative impact on the mental health of the general population (Dong & Bouey, 2020). Second, currently, most of the infected people in Shanghai are asymptomatic or mild and have been vaccinated (Accorsi et al., 2022; Zhang et al., 2022), and the daily consumable supply and medical protection in shelter hospitals are relatively adequate. Thus, people may feel less panic about the epidemic. These factors could be protective factors to mitigate the adverse effects of the pandemic on mental health. Nevertheless, the impact on mental health or sleep quality due to changes in the living environment and environmental noise during isolation cannot be ignored (Andrillon et al., 2016).

Stratified analysis by marital status showed that there was no significant difference in the prevalence of depression and anxiety among the different marital status groups, but the unmarried group exhibited lower rumination scores and better sleep status. Furthermore, the present results demonstrated that the degree of rumination was positively correlated with the severity of depression and anxiety symptoms, respectively. Generally, rumination mainly reflects the individual's psychological coping style in the face of negative life events (Beevers, 2005; Nolen‐Hoeksema et al., 2008). Individuals with high levels of rumination are more likely to develop anxiety and depression symptoms (Harding & Mezulis, 2017; Liu et al., 2017).

The better mental health status of the unmarried people in this study suggests that on the one hand they are relatively young and do not have family burdens after marriage (such as children's education, spousal relationships, economic pressure, and household affairs) (Bultmann et al., 2020; Lee et al., 2021). Moreover, unmarried individuals may actively adopt mobile games or indoor entertainment to manage negative pressure during the period of quarantine (Berryman et al., 2018; Pretorius et al., 2019). These factors may facilitate the resilience of the unmarried group to reduce susceptibility to mental health distress (Mohler‐Kuo et al., 2021). This viewpoint was further supported by the results that individuals aged 18–35 years displayed lower levels of anxiety and better sleep quality in the present study.

To evaluate the results of the present study appropriately, some limitations should be considered. First, the present study is an anonymous online questionnaire survey, and the respondents were relatively young and had high levels of education; therefore, these findings may not be generalizable. Second, this survey is a cross‐sectional study and lacks follow‐up data. The depression/anxiety symptoms were based on self‐rating scales rather than face‐to‐face interviews by psychiatrists. Therefore, it can only indicate that the participants have some degree of anxiety or depression, but the diagnosis has not been confirmed. A follow‐up study is needed to assess the long‐term impact of COVID‐19 on mental health (Ceban et al., 2022; Yelin et al., 2020). Finally, because this online survey requires the use of smartphones, it did not include adolescents and the elderly individuals over 60 years old. Additionally, the survey subjects of this study were mainly asymptomatic or mildly infected people with the Omicron variant of COVID‐19, which does not represent the mental health status of severely/critically infected people.

## 5 CONCLUSION

The survey found that women are more likely to report anxiety and depression symptoms than men, and middle‐aged people exhibited prominent anxiety symptoms. In addition, younger, unmarried people showed less mental health distress (anxiety, depression, and sleep disturbance). Nonadaptive rumination may exacerbate susceptibility to anxiety‐depressive symptoms due to pernicious repercussions for mental health. Although the somatic harm of the Omicron variant to the population has decreased compared with before, it is still imperative to identify of populations with high risk and enhance psychological support during the pandemic.

## AUTHOR CONTRIBUTIONS

Drs Hou and Yuan designed the study; Drs Hou, Huang, Ma, Feng, Fu, Li and Yuan collected the data; Drs Hou and Feng performed the literature search and statistical analysis; Hou wrote the first draft of the manuscript. All authors provided major advice and revision in the drafting of the manuscript. Dr Hou and Yuan had full access to the integrated data in the study and takes responsibility for the analysis of the data.

## FUNDING

This study was supported by the Natural Science Foundation of Jiangsu Province (Hou, NO. BK20201270), the National Natural Science Foundation of China (Yuan, NO. 81971277).

## CONFLICT OF INTEREST

No disclosure of conflict of interest.

### PEER REVIEW

The peer review history for this article is available at: https://publons.com/publon/10.1002/brb3.2803

## ETHICAL STANDARDS

The authors state that this work complies with the ethical standards of the relevant national and institutional committees on human experimentation and with the Helsinki Declaration of 1975, as revised in 2008.

## Data Availability

Author will provide data if required.
